# Investigating highly replicated asthma genes as candidate genes for allergic rhinitis

**DOI:** 10.1186/1471-2350-14-51

**Published:** 2013-05-10

**Authors:** Anand Kumar Andiappan, Daniel Nilsson, Christer Halldén, Wang De Yun, Torbjörn Säll, Lars Olaf Cardell, Chew Fook Tim

**Affiliations:** 1 Department of Biological Sciences, National University of Singapore, Science Drive 4, Singapore, 117543, Singapore; 2 Singapore Immunology Network (SIgN), Agency for Science, Technology and Research (A*STAR) 8A Biomedical Grove, Singapore, 138648, Singapore; 3 Division of ENT Diseases, CLINTEC, Karolinska Institutet, Huddinge, Sweden; 4 Kristianstad University, Section Biomedicine, Kristianstad, Sweden; 5 Department of Otolaryngology, National University of Singapore, 10 Lower Kent Ridge Road, Singapore, 119260, Singapore; 6 Department of Cell and Organism Biology, Lund University, Lund, Sweden

**Keywords:** Allergic rhinitis, Association, Asthma, Case–control, Replication

## Abstract

**Background:**

Asthma genetics has been extensively studied and many genes have been associated with the development or severity of this disease. In contrast, the genetic basis of allergic rhinitis (AR) has not been evaluated as extensively. It is well known that asthma is closely related with AR since a large proportion of individuals with asthma also present symptoms of AR, and patients with AR have a 5–6 fold increased risk of developing asthma. Thus, the relevance of asthma candidate genes as predisposing factors for AR is worth investigating. The present study was designed to investigate if SNPs in highly replicated asthma genes are associated with the occurrence of AR.

**Methods:**

A total of 192 SNPs from 21 asthma candidate genes reported to be associated with asthma in 6 or more unrelated studies were genotyped in a Swedish population with 246 AR patients and 431 controls. Genotypes for 429 SNPs from the same set of genes were also extracted from a Singapore Chinese genome-wide dataset which consisted of 456 AR cases and 486 controls. All SNPs were subsequently analyzed for association with AR and their influence on allergic sensitization to common allergens.

**Results:**

A limited number of potential associations were observed and the overall pattern of *P-*values corresponds well to the expectations in the absence of an effect. However, in the tests of allele effects in the Chinese population the number of significant *P-*values exceeds the expectations. The strongest signals were found for SNPs in *NPSR1* and *CTLA4*. In these genes, a total of nine SNPs showed *P-*values <0.001 with corresponding *Q-*values <0.05. In the *NPSR1* gene some *P-*values were lower than the Bonferroni correction level. Reanalysis after elimination of all patients with asthmatic symptoms excluded asthma as a confounding factor in our results. Weaker indications were found for *IL13* and *GSTP1* with respect to sensitization to birch pollen in the Swedish population.

**Conclusions:**

Genetic variation in the majority of the highly replicated asthma genes were not associated to AR in our populations which suggest that asthma and AR could have less in common than previously anticipated. However, *NPSR1* and *CTLA4* can be genetic links between AR and asthma and associations of polymorphisms in *NPSR1* with AR have not been reported previously.

## Background

Allergic rhinitis (AR) is a major chronic respiratory disease and the most common allergic disorder with a worldwide prevalence of 10-25% [1]. It has long been recognized that the development of AR is dependent on interactions between genetic and environmental factors and that the genetic factors play a major role with an estimated heritability for AR as high as 70-90% [2,3]. A large number of studies have identified more than 100 single nucleotide polymorphisms (SNPs) associated with AR, but few of them have been successfully replicated. The general reproducibility of a majority of these AR associations was found to be low in a previous study [4]. Only one genome-wide association study (GWAS) has been performed to identify genetic variants specifically for AR [5]. In that study, no associations were detected at a genome-wide significance level, and only two at a suggestive significance level. In addition, a genome-wide meta-analysis based on self-reported AR identified few genetic variants in spite of analyzing 2.2 million SNPs in close to 4000 AR cases and 9000 controls [6]. Only one locus reached genome-wide significance and six suggestive loci were identified. Comparing these two studies there were no significant association signals in common. In addition, no association signals of earlier linkage and candidate gene association studies coincide with any of the loci identified in the GWAS and the meta-study. This is not surprising, since the replication of different genotype-phenotype associations has in general proven to be much more difficult than initially appreciated [7]. Experience gathered in several different diseases indicates that there is a positive correlation between the ability to replicate previous associations and the size and number of previous studies. Thus, in diseases where many large previous association studies have been performed replication is often more successful.

It is well known that asthma is closely related to AR since a large proportion of individuals with asthma also present symptoms of AR, and patients with AR have a 5–6 fold increased risk of developing asthma [8-10]. Asthma genetics has been extensively studied and many genes have been associated with the development or severity of this disease. Ober and Hoffjan (2006) listed 118 genes that had been reported to be associated with asthma or atopy-related phenotypes [11]. Of these genes, 25 were positively associated with asthma in six or more independent studies and were thus highly implicated as true susceptibility genes for asthma-related phenotypes. The original associations were identified in many different types of studies; linkage, positional cloning and candidate gene association studies and were subsequently replicated mostly by association studies. Several studies have investigated the reproducibility of asthma candidate gene associations. One study by Rogers et al. (2009) investigated 160 associated SNPs from 39 genes from an Illumina 550k array in 422 families and successfully replicated 10 SNPs in six genes [12]. At the level of the gene they found additional support for association in 15 of the 39 genes but none were significant after adjustment for multiple comparisons. In a consortium-based GWAS investigating 10,365 asthma patients with 16,110 controls a total of seven loci with genome-wide significance were identified [13]. None of the implicated genes overlapped with the genes identified in the study by Rogers et al. [12]. A meta-analysis of GWAS in ethnically diverse North American populations identified five susceptibility loci, four of whom at previously reported loci [14]. Also this study was very large and analyzed 5,416 patients with replication in 12,649 patients. The combined results of the many previous association studies for asthma illustrate the challenges in the search and replication of risk factors for asthma.

Due to the close relationship between the occurrence of asthma and AR we hypothesize that these two phenotypes have genetic risk factors in common. The present study was thus designed to investigate if SNPs in highly replicated asthma genes as reported in the Ober and Hoffjan study [11] are associated with the occurrence of AR.

## Methods

### Subjects

The Swedish study population consists of 246 AR patients (108 female, 138 male) and 431 control individuals (185 female, 246 male) and was recruited from southern Sweden in 2003–2009. All subjects are unrelated and of western European origin, with both parents born in Sweden. The diagnosis of birch and/or grass pollen induced AR was based on a positive history of AR for at least two years and a positive skin prick test (SPT) or Phadiatop test with at least class two to birch and/or timothy grass pollen. A total of 59% of the patients showed a positive SPT for both allergens. All patients were classified as having severe symptoms i.e., nasal itching, sneezing, rhinorrhea and nasal congestion during pollen season and they had all been treated with antihistamines and nasal steroids during pollen seasons previous years. Control individuals had no history of AR or any other atopic disease and had a negative SPT or Phadiatop test. Genomic DNA was extracted from blood collected in EDTA using QIAamp DNA Blood Maxi or Mini kits (Qiagen, Hilden, Germany) and DNA concentrations determined by fluorometry using PicoGreen (Molecular Probes, Invitrogen, Eugene, OR, USA). The Swedish study population has previously been analyzed in several AR studies [4,15-17]. The Singapore Chinese population consists of 448 AR patients (250 female, 198 male) and 462 control individuals (337 female and 125 male) and was collected in Singapore between 2008 and 2010. The study population is of Chinese origin, residence of Singapore and all subjects are unrelated to one another. The diagnosis of dust mite induced AR was based on interviews of medical history using a standardized questionnaire and SPTs performed using a panel of common allergens in Singapore including the house dust mite *Dermatophagoides pteronyssinus* and *Blomia tropicalis*. A total of 89% of the patients showed a positive SPT for both allergens. AR is thus diagnosed based on the presence of atopic status and typical AR symptoms as defined by the ARIA 2008 guidelines i.e., two or more AR symptoms (nasal congestion, rhinorrhea, nasal itching, sneezing) persisting for four or more days a week during the past year. In this study, none of the patients suffered from severe asthma and less than 10% had moderate asthma with continuous medication. Control individuals had no history of AR or any other atopic disease and had a negative SPT. More detailed phenotypic characteristics of the Singapore Chinese population have been described previously [5]. Genomic DNA was extracted from buccal cells obtained from a mouthwash of 0.9% saline solution following a standardized protocol [18]. Samples were quantified in triplicate on the Nanodrop (Thermo Fisher Scientific Inc, Wilmington, DE, USA). In both populations, SPT were performed with saline buffer as negative and histamine chloride as positive controls. A wheal reaction diameter of more than three mm was considered a positive SPT response. SPT was only performed if the AR cases had not taken any anti-allergic drugs for at least three days prior to the test. Atopy is defined as a positive SPT reaction to either one of allergens. The study was approved by the Ethics Committee of the Medical Faculty, Lund University and the Institutional Review Board of National University of Singapore and written informed consent was obtained from all subjects. This study is also in compliance with the Helsinki declaration.

### Genotyping

A total of 21 genes (*IL4*, *IL13*, *CD14*, *MS4A2 (FCERB1)*, *IL4R (IL4RA)*, *ADAM33*, *GSTM1*, *IL10*, *CTLA4*, *SPINK5*, *LTC4S*, *NPSR1 (GPR154)*, *NOD1 (CARD4)*, *SCGB1A1 (CC16)*, *GSTP1*, *STAT6*, *NOS1*, *CCL5*, *TBXA2R*, *ADRB2* and *TGFB1*) were selected from a compilation of 25 genes reported to be associated with asthma phenotypes in six or more unrelated independent studies [11]. SNPs of the 21 genes were analyzed for association with AR whereas no SNPs were analyzed for *HLA-DRB1*, *HLA-DQB1*, *TNF*, and *LTA* as they are located at the HLA locus. Since this study investigates asthma genes for their eventual contribution also to the AR phenotype, we use the gene as the replication unit and not the individual SNPs. For genotyping in the Swedish study population, HapMap (release 24) data were used to identify haplotype-tagging SNPs (r^2^ cut off =0.8, and minor allele frequency cut off =0.2) for each of the 21 genes. Non-synonymous SNPs reported in dbSNP or HapMap with minor allele frequencies >5% was added to this selection. Genotypes were determined using the Sequenom MassARRAY MALDI-TOF system. Assay design was made using the MassARRAY Assay Design ver. 2.0 software (Sequenom Inc, San Diego, CA, USA) and primers were obtained from Metabion GmbH (Martinsried, Germany). A total of 192 SNPs with a total genotyping rate of 98.4% were analyzed for association with AR in 246 patients and 431 controls. Whole genome genotyping in the Singapore Chinese population was performed using the Illumina HumanHap 550 k BeadChip version 3 (Illumina, San Diego, CA, USA) at the Genome Institute of Singapore as described earlier [5]. Of the 21 genes initially selected, three genes (*LTC4S*, *NOD1* and *GSTM1*) had no SNPs in the Illumina Human Hap 550k panel while *CCL5* had one SNP but was filtered out after quality control for SNPs. A total of 413 SNPs from 17 unique genes with a total genotyping rate of 98.4% were analyzed for association with AR in 448 patients and 462 controls. Additional samples (921 AR patients, 390 controls) from the Singapore Chinese cohort were genotyped for rs324981, using a Taqman-assay. Reactions were carried out according to the manufacturer’s protocol using an ABI PRISM 7900HT and the genotypes determined using SDS 2.4 software (Applied Biosystems, Foster City, CA, USA).

### Statistical analysis

Statistical analyses were made using R statistical software [19] and PLINK v1.07 [20]. Genotype frequencies were calculated and tested for Hardy-Weinberg equilibrium in both cases and controls. Allele and genotype frequencies were then investigated for association with AR using a χ^2^-homogeneity test. Odds ratios and 95% confidence intervals were estimated by using the most common allele as the referent and are reported for each minor allele. Associations between SPT-response and genotype were analyzed using Kruskal-Wallis rank sum test. False discovery rate was quantified using the *Q-*value introduced by Storey [21] and calculated using the software QVALUE (ver.1.0).

## Results

### Association with AR phenotype in the Swedish population

A total of 192 SNPs from 21 genes were genotyped and analyzed for association with AR in 246 patients and 431 controls. For each SNP two tests of association were made, one of allele frequencies and one of genotype frequencies. Table 1 shows the results of the association analysis for SNPs with uncorrected *P-*values <0.05 and the corresponding *Q-*values and ORs (for complete results see Additional file 1: Table S1). In the tests of allele frequencies there were two *P-*values <0.01 and 11 *P*-values *<*0.05. In the tests of genotype frequencies there were three *P-*values <0.01 and eight *P*-values *<*0.05. None of the indicated SNPs had *Q-*values <0.1. In the absence of any association, the expected numbers of *P-*values <0.01 and <0.05 are 1.9 and 9.6, respectively, in each of the test categories. Thus, the results fit very well the expected pattern of *P-*values in the absence of any effects.

**Table 1:** Minor allele frequencies (MAF) and P-values for Hardy-Weinberg (HW) and association tests for SNPs with P<0.05 in the Swedish population

**Gene**	**SNP ID**	**Chromosome position**		**Study MAF**	**HW test**	**Association test**^**†**^				**OR**	**95% CI**	
						**Allele**		**Genotype**			**Lower**	**Upper**
*IL10*	rs3021094	1	206944952	0.092	0.16	0.020	(0.72)	0.078	(0.92)	1.6	1.1	2.2
*NOD1*	rs4363092	7	30503938	0.18	0.36	0.050	(0.72)	0.045	(0.92)	1.3	1.0	1.8
*NOD1*	rs4720003	7	30508992	0.18	0.37	0.041	(0.72)	0.033	(0.92)	1.3	1.0	1.8
*NPSR1*	rs2022142	7	34705950	0.10	1.0	0.035	(0.72)	0.056	(0.92)	1.5	1.0	2.1
*NPSR1*	rs1379925	7	34715128	0.091	1.0	0.021	(0.72)	0.031	(0.92)	1.6	1.1	2.3
*NPSR1*	rs1379923	7	34717238	0.25	0.41	0.045	(0.72)	0.11	(0.92)	0.8	0.6	1.0
*NPSR1*	rs17788770	7	34793991	0.10	0.68	0.083	(0.72)	0.035	(0.92)	0.7	0.5	1.0
*NPSR1*	rs17789834	7	34871328	0.19	0.32	0.011	(0.70)	0.039	(0.92)	1.4	1.1	1.9
*SCGB1A1*	rs11231085	11	62190448	0.36	0.74	0.037	(0.72)	0.0049	(0.58)	1.3	1.0	1.6
*CCL5*	rs1065341	17	34198593	0.040	0.41	0.027	(0.72)	0.085	(0.92)	1.8	1.1	3.2
*CCL5*	rs3817655	17	34199641	0.17	0.12	0.0028	(0.29)	0.0088	(0.58)	1.6	1.2	2.1
*CCL5*	rs2107538	17	34207780	0.17	0.27	0.0021	(0.29)	0.0069	(0.58)	1.6	1.2	2.1
*TBXA2R*	rs3786989	19	3604004	0.93	0.25	0.038	(0.72)	0.086	(0.92)	0.6	0.4	1.0

†Association with AR estimated using a χ2-homogeneity test with Q-values in parenthesis calculated according to Storey (2002).

Odds ratio (OR) and 95% confidence interval were estimated by using the most common allele as the referent and are reported for each minor allele.

### Association with SPT response in the Swedish population

A Kruskal-Wallis rank sum test was used to investigate the relationship between genotype and degree of sensitization to birch and timothy grass in AR-patients. A total of 24 SNPs had *P-*values <0.05 in the tests of SPTs for birch or timothy grass (Table 2 and Additional file 1: Table S1). In the tests of birch there were three *P-*values <0.001, four at *P*<0.01 and 10 at *P*<0.05 and in the test of timothy grass there were one *P-*value <0.01 and 16 at *P*<0.05. The *Q-*values of the three SNPs with *P<*0.001 for birch (rs1138272 in *GSTP1*, rs20541 and rs848 in *IL13*) were approximately 0.04 and all other *Q-*values were >0.1. The lowest *P-*value (rs1138272) is below the level for a Bonferroni corrected *P-*value of 0.05, i.e. 0.00022 vs. 0.00026. Although the overall distribution of *P-*values is close to the expectation under the assumption of absence of any association, the three *P-*values <0.001 can be considered an indication of an effect that warrants further investigation.

**Table 2:** Association of SNPs with P<0.05 for sensitization to allergens in the Swedish population

**Gene**	**SNP ID**	**Chromosome position**		**Kruskal-Wallis test**			
				**Birch**		**Timothy**	
*IL10*	rs3024498	1	206941529	0.27	(0.95)	0.0094	(0.31)
*IL10*	rs3024492	1	206944112	0.17	(0.87)	0.025	(0.36)
*IL13*	rs20541	5	131995964	0.00040	(0.038)	0.58	(0.77)
*IL13*	rs848	5	131996500	0.00067	(0.043)	0.56	(0.77)
*IL4*	rs2243248	5	132008644	0.0044	(0.21)	0.52	(0.76)
*IL4*	rs2070874	5	132009710	0.051	(0.87)	0.016	(0.31)
*IL4*	rs2227284	5	132012725	0.015	(0.57)	0.18	(0.59)
*IL4*	rs2243266	5	132013789	0.046	(0.87)	0.014	(0.31)
*IL4*	rs2243288	5	132017944	0.046	(0.87)	0.013	(0.31)
*SPINK5*	rs4357026	5	147457939	0.77	(0.99)	0.048	(0.45)
*SPINK5*	rs9325073	5	147498652	0.90	(0.99)	0.020	(0.31)
*SPINK5*	rs1422993	5	147503820	0.96	(0.99)	0.015	(0.31)
*SPINK5*	rs4263489	5	147516195	0.049	(0.87)	0.61	(0.77)
*LTC4S*	rs730012	5	179220638	0.042	(0.87)	0.57	(0.77)
*NPSR1*	rs323917	7	34741643	0.048	(0.87)	0.35	(0.63)
*NPSR1*	rs12534369	7	34804709	0.64	(0.96)	0.033	(0.43)
*NPSR1*	rs17170017	7	34874209	0.54	(0.95)	0.011	(0.31)
*N.D.*	rs10897270	11	62183007	0.90	(0.99)	0.012	(0.31)
*GSTP1*	rs1138272	11	67353579	0.00022	(0.038)	0.20	(0.61)
*NOS1*	rs6490121	12	117708195	0.33	(0.95)	0.036	(0.44)
*NOS1*	rs12578547	12	117763347	0.091	(0.87)	0.018	(0.31)
*NOS1*	rs3782218	12	117771511	0.96	(0.99)	0.046	(0.45)
*NOS1*	rs17509231	12	117794323	0.43	(0.95)	0.011	(0.31)
*IL4R*	rs1029489	16	27376217	0.46	(0.95)	0.039	(0.44)

Association between genotype and sensitization to allergens was analyzed using a Kruskal-Wallis rank sum test. False discovery rate was quantified using the q-value introduced by Storey(2002) and are given in parenthesis.

### Association with AR phenotype in the Chinese population

Of the 21 genes initially selected, a total of 413 SNPs from 17 genes were extracted from the Illumina Human Hap 550 k panel and tested for association with AR in the Singapore Chinese population. Just as in the Swedish population two association tests were made, one at the allele and one at the genotype level. Table 3 shows the results of the association analysis for SNPs with uncorrected *P-*values <0.05 and the corresponding *Q-*values and ORs (for complete results see Additional file 2: Table S2). A total of 50 SNPs had *P-*values <0.05. In the association test of alleles there were nine *P-*values <0.001, 18 at *P*< 0.01 and a total of 48 at *P*<0.05. All nine SNPs with *P-*values <0.001 had *Q-*values <0.05 and corresponds to rs10270663, rs324389, rs324957, rs10278663, rs324396, rs10267134, rs324987 in *NPSR1/AAA1*, and rs231804 and rs231735 in *CTLA4*. In the tests of association at the genotype level, the four lowest *P-*values were <0.001with *Q-*values <0.05 and coincided with the lowest *P-*values in the allele test. In addition, another nine *P-*values were <0.01 and five of them had *Q-*values <0.1. Of these five, four also coincide with the nine SNPs with *P*<0.001 in the allele test. In total, there were 30 *P-*values <0.05. In the absence of any association, the expected number of *P-*values <0.001, <0.01 and <0.05 are 0.4, 4.1 and 20.7, respectively, in each of the test categories. Thus, the observed numbers of significant *P-*values exceeds the expectation in the absence of any association for both the allele and the genotype tests. In the tests of allele effects, three *P-*values were lower than 0.00012 which is the Bonferroni limit at *P*=0.05 within each category. The lowest *P-*value, 0.000068, is larger than 0.00003 equal to the global Bonferroni limit for all tests taken together, but of the same order of magnitude. Thus, these results give a strong indication of a genetic effect of *NPSR1/AAA1* and *CTLA4* on the occurrence of AR, i.e. these genes can be considered strong candidates for future analyses.

**Table 3:** Minor allele frequencies (MAF) and P-values for Hardy-Weinberg (HW) and association tests for SNPs with P<0.05 in the Chinese population

**Gene**	**SNP ID**	**Chromosome position**		**Study MAF**	**HW test**	**Association test**				**OR**	**95% CI**	
						**Allele**		**Genotype**			**Lower**	**Upper**
*CTLA4*	rs231735	2	204402121	0.23	1.0	0.00090	(0.037)	0.0038	(0.12)	1.44	1.16	1.79
*CTLA4*	rs231804	2	204416891	0.22	0.93	0.00060	(0.028)	0.0025	(0.090)	1.46	1.18	1.82
*CTLA4*	rs1024161	2	204429997	0.34	0.57	0.0088	(0.21)	0.020	(0.34)	1.29	1.07	1.56
*CTLA4*	rs926169	2	204430997	0.34	0.89	0.0059	(0.20)	0.016	(0.29)	1.31	1.08	1.58
*CTLA4*	rs733618	2	204439189	0.40	1.0	0.012	(0.22)	0.030	(0.42)	0.79	0.66	0.95
*CTLA4*	rs231726	2	204449111	0.40	0.74	0.016	(0.22)	0.041	(0.50)	1.25	1.04	1.51
*CTLA4*	rs6748358	2	204465150	0.28	0.39	0.0077	(0.21)	0.0067	(0.18)	1.31	1.08	1.61
*CTLA4*	rs10197319	2	204471289	0.22	0.86	0.013	(0.22)	0.0058	(0.17)	1.31	1.06	1.63
*CTLA4*	rs3096851	2	204472127	0.40	0.90	0.015	(0.22)	0.028	(0.41)	1.26	1.05	1.51
*CTLA4*	rs3116504	2	204477299	0.40	0.90	0.015	(0.22)	0.028	(0.41)	1.26	1.05	1.51
*SPINK5*	rs7707803	5	147368427	0.33	0.23	0.021	(0.27)	0.077	(0.57)	1.25	1.03	1.52
*SPINK5*	rs7725292	5	147368581	0.33	0.28	0.017	(0.22)	0.061	(0.51)	1.27	1.04	1.54
*SPINK5*	rs10477360	5	147384474	0.50	0.90	0.037	(0.34)	0.066	(0.52)	0.82	0.69	0.99
*SPINK5*	rs12332673	5	147387572	0.12	0.17	0.045	(0.36)	NA	(NA)	0.75	0.56	0.99
*SPINK5*	rs11948836	5	147393693	0.12	0.17	0.039	(0.34)	NA	(NA)	0.74	0.56	0.99
*SPINK5*	rs17774892	5	147395161	0.12	0.88	0.035	(0.34)	NA	(NA)	0.74	0.55	0.98
*SPINK5*	rs10463396	5	147395535	0.20	1.0	0.016	(0.22)	0.053	(0.50)	0.76	0.60	0.95
*SPINK5*	rs17107650	5	147396591	0.12	0.17	0.039	(0.34)	NA	(NA)	0.74	0.56	0.99
*SPINK5*	rs1422982	5	147400217	0.20	1.00	0.016	(0.22)	0.053	(0.50)	0.76	0.60	0.95
*SPINK5*	rs17107673	5	147401681	0.12	0.29	0.041	(0.34)	NA	(NA)	0.74	0.56	0.99
*SPINK5*	rs4472254	5	147433830	0.20	0.69	0.016	(0.22)	0.044	(0.50)	0.76	0.60	0.95
*SPINK5*	rs7724165	5	147445445	0.50	0.80	0.027	(0.30)	0.054	(0.50)	1.23	1.02	1.47
*SPINK5*	rs4519913	5	147452004	0.50	0.85	0.024	(0.27)	0.057	(0.50)	0.81	0.68	0.97
*ADRB2*	rs11742519	5	148218501	0.45	0.90	0.039	(0.34)	0.056	(0.50)	1.21	1.01	1.45
*NPSR1*	rs411323	7	34668233	0.16	0.81	0.037	(0.34)	0.095	(0.62)	1.31	1.02	1.69
*NPSR1/AAA1*	rs10081183	7	34706738	0.45	0.66	0.0066	(0.21)	0.023	(0.36)	0.78	0.65	0.93
*NPSR1/AAA1*	rs1345267	7	34714584	0.46	0.31	0.012	(0.22)	0.036	(0.48)	0.79	0.66	0.95
*NPSR1/AAA1*	rs1419791	7	34722086	0.48	0.85	0.0096	(0.21)	0.010	(0.21)	1.27	1.06	1.52
*NPSR1/AAA1*	rs1419791	7	34722086	0.48	0.85	0.0096	(0.21)	0.010	(0.21)	1.27	1.06	1.52
*NPSR1/AAA1*	rs324374	7	34723121	0.48	0.90	0.0095	(0.21)	0.010	(0.21)	1.27	1.06	1.52
*NPSR1/AAA1*	rs324389	7	34744239	0.46	0.66	0.00010	(0.014)	0.00055	(0.044)	0.70	0.58	0.84
*NPSR1/AAA1*	rs10270663	7	34752923	0.46	0.52	0.000068	(0.014)	0.00038	(0.044)	0.69	0.58	0.83
*NPSR1/AAA1*	rs324396	7	34756648	0.42	1.0	0.00035	(0.021)	0.0012	(0.065)	1.40	1.16	1.68
*NPSR1/AAA1*	rs324957	7	34767897	0.43	0.61	0.00011	(0.014)	0.00052	(0.044)	1.44	1.20	1.73
*NPSR1/AAA1*	rs10267134	7	34769628	0.34	0.89	0.00039	(0.021)	0.0014	(0.065)	0.71	0.58	0.86
*NPSR1/AAA1*	rs10278663	7	34774996	0.34	0.83	0.00032	(0.021)	0.0012	(0.065)	0.70	0.58	0.85
*NPSR1*	rs324987	7	34787953	0.44	0.34	0.00021	(0.019)	0.00049	(0.044)	1.41	1.18	1.70
*NPSR1*	rs17199888	7	34830864	0.33	0.89	0.0022	(0.083)	0.0075	(0.18)	0.74	0.61	0.90
*NPSR1*	rs1419868	7	34834082	0.25	0.018	0.013	(0.22)	0.054	(0.50)	1.31	1.06	1.61
*GSTP1*	rs614080	11	67103863	0.28	0.44	0.40	(0.80)	0.050	(0.50)	1.09	0.89	1.33
*NOS1*	rs884847	12	116207996	0.15	0.90	0.26	(0.75)	0.043	(0.50)	1.01	0.90	1.50
*NOS1*	rs532967	12	116216722	0.20	0.55	0.032	(0.34)	0.0024	(0.090)	1.03	1.02	1.62
*NOS1*	rs545654	12	116261432	0.32	0.47	0.019	(0.24)	0.043	(0.50)	1.19	0.65	0.96
*NOS1*	rs693534	12	116269101	0.28	0.64	0.0094	(0.21)	0.014	(0.29)	0.84	0.62	0.94
*NOS1*	rs3782221	12	116280264	0.49	0.31	0.024	(0.27)	0.047	(0.50)	1.08	0.67	0.97
*NOS1*	rs11068466	12	116320260	0.17	0.73	0.023	(0.27)	0.016	(0.29)	0.91	0.59	0.96
*NOS1*	rs10774914	12	116331323	0.12	0.56	0.042	(0.34)	0.10	(0.62)	1.13	1.01	1.78
*IL4R*	rs3024535	16	27259622	0.16	0.023	0.034	(0.34)	NA	(NA)	1.31	1.02	1.68
*IL4R*	rs3024585	16	27267345	0.38	0.12	0.034	(0.34)	0.088	(0.62)	0.82	0.68	0.99
*IL4R*	rs2074570	16	27282658	0.07	0.63	0.040	(0.34)	NA	(NA)	1.45	1.02	2.07

†Association with AR estimated using a χ2-homogeneity test with Q-values in parenthesis calculated according to Storey (2002).

Odds ratio (OR) and 95% confidence interval were estimated by using the most common allele as the referent and are reported for each minor allele.

In addition, we investigated the functional *NPSR1* coding variant rs324981 (Ile107Asn), which is in complete linkage disequilibrium with rs324987 (*P*-value for association =0.00021, OR=1.41, see Table 3). A TaqMan assay was used to determine the rs324981 genotypes of additional individuals of the Singapore Chinese cohort (921 AR patients, 390 controls). The *P-*value of association was 0.0070 with an odds ratio (OR) of 1.14 for the heterozygous genotype and an increased OR of 1.65 for the homozygous genotype in comparison with the reference genotype. Thus, both analyses support the involvement of *NPSR1* in AR.

Since genetic variation of all genes tested for association with AR in the present study has previously been associated with asthma and since patients with AR have an increased incidence of asthma, we investigated asthma as a confounding factor for our results. Since the strongest associations are detected in the Chinese population, we excluded all Chinese patients with any asthmatic symptoms and repeated the association analysis for this population (Additional file 3: Table S3). Comparing the significant association results before and after elimination of AR patients with asthma shows that: 1) The elimination of 144 patients with asthma out of 448 (32%) result in a general increase in *P*-values corresponding to the loss of power due to a smaller sample size, 2) the nine SNPs with *P*-values <0.001 detected in the initial analysis of all 448 AR patients, all showed *P*-values ≤0.01 after elimination of the 144 patients, 3) these SNPs also showed very small changes in their ORs (<0.08), 4) most ORs were highly similar before and after elimination of the 144 patients, the exception being five SNPs in the *NOS1* gene that may indicate confounding due to asthma.

### Association with SPT response in the Chinese population

A Kruskal-Wallis rank sum test was used to investigate the relationship between genotype and degree of sensitization to *D. pteronyssinus* and *B. tropicalis* in AR-patients. All tests of the SPT with an uncorrected *P-*value <0.05 are shown in Table 4 (for complete results see Additional file 2: Table S2). A total of 44 SNPs had *P-*values <0.05 in the tests of *D. pteronyssinus* and *B. tropicalis*. In the tests of *B. tropicalis* there were six *P-*values <0.01 and a total of 24 *P-*values <0.05. In the tests of *D. pteronyssinus* one *P-*value was <0.001, six were <0.01 and a total of 22 were <0.05. None of the indicated SNPs had corresponding *Q-*values <0.1. Thus, the tests of the SPT response conformed well to the expectations under independence and give therefore no indication of any association with sensitization of the tested allergens.

**Table 4:** Association of SNPs with P<0.05 for sensitization to allergens in the Chinese population

**Gene**	**SNP ID**	**Chromosome position**		**Kruskal-Wallis test**			
				***B.tropicalis***		***D. pteronyssinus***	
*SPINK5*	rs10477360	5	147384474	0.027	(0.65)	0.69	(1.0)
*ADRB2*	rs2163752	5	148125331	0.027	(0.65)	0.054	(0.92)
*ADRB2*	rs30306	5	148132557	0.028	(0.65)	0.074	(0.97)
*ADRB2*	rs30325	5	148143517	0.27	(0.82)	0.010	(0.52)
*ADRB2*	rs30328	5	148146640	0.28	(0.82)	0.025	(0.75)
*ADRB2*	rs30330	5	148148525	0.32	(0.82)	0.027	(0.75)
*ADRB2*	rs9285673	5	148153121	0.049	(0.78)	0.90	(1.0)
*ADRB2*	rs10075995	5	148273622	0.53	(0.82)	0.014	(0.52)
*NPSR1/AAA1*	rs2058163	7	34301616	0.049	(0.78)	0.025	(0.75)
*NPSR1/AAA1*	rs1419842	7	34321625	0.41	(0.82)	0.031	(0.75)
*NPSR1/AAA1*	rs2392268	7	34380952	0.44	(0.82)	0.031	(0.75)
*NPSR1/AAA1*	rs6947789	7	34417405	0.018	(0.65)	0.83	(1.0)
*NPSR1/AAA1*	rs736295	7	34417742	0.054	(0.79)	0.039	(0.75)
*NPSR1*	rs2530545	7	34663665	0.94	(0.90)	0.040	(0.75)
*NPSR1*	rs11761197	7	34666914	0.78	(0.90)	0.013	(0.52)
*NPSR1*	rs1379928	7	34667814	0.51	(0.82)	0.0098	(0.52)
*NPSR1*	rs2609224	7	34672931	0.62	(0.85)	0.011	(0.52)
*NPSR1*	rs2609220	7	34676579	0.68	(0.86)	0.0067	(0.52)
*NPSR1*	rs2531841	7	34686074	0.81	(0.90)	0.012	(0.52)
*NPSR1*	rs1419837	7	34692064	0.88	(0.90)	0.018	(0.62)
*NPSR1*	rs1419779	7	34779833	0.21	(0.82)	0.0011	(0.15)
*NPSR1*	rs324978	7	34780857	0.20	(0.82)	0.00098	(0.15)
*NPSR1*	rs1859409	7	34906409	0.049	(0.78)	0.83	(1.0)
*NPSR1*	rs4723388	7	34909345	0.0056	(0.51)	0.52	(0.98)
*NPSR1*	rs1186717	7	34937434	0.33	(0.82)	0.036	(0.75)
*NPSR1*	rs1637673	7	34964375	0.15	(0.82)	0.0081	(0.52)
*NPSR1*	rs4236340	7	34976719	0.0061	(0.51)	0.46	(0.98)
*NPSR1*	rs328902	7	34987368	0.41	(0.82)	0.038	(0.75)
*NPSR1*	rs328906	7	34990440	0.0080	(0.51)	0.58	(0.98)
*NPSR1*	rs2023328	7	34998155	0.0053	(0.51)	0.60	(0.98)
*NPSR1*	rs329240	7	35024965	0.41	(0.82)	0.038	(0.75)
*MS4A2*	rs540170	11	59636614	0.049	(0.78)	0.98	(1.0)
*MS4A2*	rs581133	11	59638882	0.043	(0.78)	0.99	(1.0)
*NOS1*	rs1093325	12	116179703	0.031	(0.7)	0.23	(0.98)
*NOS1*	rs1004356	12	116261755	0.026	(0.65)	0.43	(0.98)
*IL4R*	rs3024585	16	27267345	0.0036	(0.51)	0.98	(1.0)
*IL4R*	rs1805011	16	27281373	0.011	(0.51)	0.076	(0.97)
*IL4R*	rs1805012	16	27281465	0.011	(0.51)	0.076	(0.97)
*IL4R*	rs1805015	16	27281681	0.022	(0.65)	0.11	(0.97)
*IL4R*	rs3024685	16	27284411	0.045	(0.78)	0.21	(0.98)
*IL4R*	rs4787956	16	27285750	0.0087	(0.51)	0.17	(0.97)
*IL4R*	rs4787426	16	27292232	0.026	(0.65)	0.37	(0.98)
*ADAM33*	rs512625	20	3596378	0.017	(0.65)	0.22	(0.98)
*ADAM33*	rs2853210	20	3606211	0.046	(0.78)	0.0010	(0.15)

Association between genotype and sensitization to allergens was analyzed using a Kruskal-Wallis rank sum test. False discovery rate was quantified using the q-value introduced by Storey(2002) and are given in parenthesis.

## Discussion

In this study, we investigated the SNP associations of well-replicated asthma candidate genes with AR in two independent populations, one Swedish and one Singapore Chinese population. Since there are inherent differences in the genetic architecture between the two populations and this study investigates asthma genes for their eventual contribution also to the AR phenotype, the gene was used as the level of replication and not the individual SNP [22]. A limited number of potential associations were observed and the overall pattern of *P-*values corresponds in general well to the expectations in the absence of an effect. However, in the tests of allele effects in the Chinese population, the number of significant *P-*values exceeds the expectations. The strongest signals were found for SNPs in *CTLA4* and *NPSR1*. In each of these genes, more than one SNP showed *P-*values <0.05 with corresponding *Q-*values <0.05. In the *NPSR1* gene some *P-*values were lower than the Bonferroni correction level indicating the existence of a true association. When comparing the results from the two populations, i.e. Table 1 vs Table 3 and Table 2 vs Table 4, it is with few exceptions different genes that show significant SNPs. This is what is expected if the significances mainly are due to chance effects generated by the multiple testing. The conclusion that there are few genes in common between AR and asthma is further strengthened by this observation. On the other hand, there is one exception to this, the *NPSR1* gene that recurs in all four tables. This observation in turn further point to this gene as the strongest candidate for being a link between AR and asthma, even if one keep in mind the fact that the *NPSR1* gene is represented by the largest number of SNPs in both populations. To further investigate this hypothesis, one SNP in *NPSR1* (rs324981) was evaluated in an independent sample of 921 AR patients and 390 controls from the Singapore Chinese population. The results (OR=1.65, *P*=0.007) further strengthen this hypothesis.

Previous studies have reported significant associations for a large region of 47 kb in the *NPSR1* gene with asthma even after Bonferroni correction for multiple comparisons (*P*<0.001). Vergara et al. [23], investigated SNPs in the *NPSR1* (*GPR154*) gene and found associations with asthma and total IgE. Furthermore, this gene has been replicated in studies of Caucasian [24-28] and Chinese populations [29], but was not replicated in a Mexican cohort of childhood asthmatics [30]. Thus, the association between variation in *NPSR1* and asthma appear to be strongly supported. Since the present study strongly indicates an association with genetic variation in the *NPSR1* gene also in AR, there is an obvious risk of asthma being a cxonfounding factor for our results. This was investigated by comparing the association results before and after elimination of patients with any symptoms of asthma. The results convincingly showed that asthma is no confounding factor for the SNPs in *NPSR1* and *CTLA4* in the Chinese population.

The present result indicates that *NPSR1* could be a genetic link between AR and asthma and associations of *NPSR1* polymorphisms with AR have not been reported prior to this.

## Conclusion

In summary, we have identified *NPSR1* and *CTLA4* as potential susceptibility genes for AR. However, these genes need to be replicated in additional populations and further characterized to elucidate their role in AR predisposition and pathogenesis. The majority of the highly replicated asthma genes were not associated with AR in our populations, which suggest that asthma and AR could be less similar at the genetic level than previously anticipated.

## Competing interests

The authors state that they have no financial or non-financial competing interests.

## Authors’ contributions

AKA, CFT, CH and LOC designed the study. AKA, TS and DN performed the data analysis and AKA, DN, CH and TS wrote the manuscript. All authors critically revised the manuscript and approved the final form of the manuscript. All authors read and approved the final manuscript.

## Pre-publication history

The pre-publication history for this paper can be accessed here:

http://www.biomedcentral.com/1471-2350/14/51/prepub

## Supplementary Material

Additional file 1: Table S1 Complete association results for the AR phenotype and allergen sensitization in the Swedish population. Click here for file

Additional file 2: Table S2 Complete association results for the AR phenotype and allergen sensitization in the Chinese population. Click here for file

Additional file 3: Table S3 Comparison of association test results before (448 patients) and after. Click here for file
